# Different Patterns of Cerebral and Muscular Tissue Oxygenation 10 Years After Coarctation Repair

**DOI:** 10.3389/fphys.2019.01500

**Published:** 2019-12-11

**Authors:** Kristof Vandekerckhove, Joseph Panzer, Ilse Coomans, Annelies Moerman, Katya De Groote, Hans De Wilde, Thierry Bové, Katrien François, Daniel De Wolf, Jan Boone

**Affiliations:** ^1^Department of Pediatric Cardiology, Ghent University Hospital, Ghent, Belgium; ^2^Department of Anesthesiology, Ghent University Hospital, Ghent, Belgium; ^3^Department of Cardiac Surgery, Ghent University Hospital, Ghent, Belgium; ^4^Department of Movement and Sports Sciences, Ghent University, Ghent, Belgium

**Keywords:** coarctation aortae, near infrared spectroscopy, exercise test, children, muscle oxygenation

## Abstract

The purpose of this study was to assess whether the lower exercise tolerance in children after coarctation repair is associated with alterations in peripheral tissue oxygenation during exercise. A total of 16 children after coarctation repair and 20 healthy control subjects performed an incremental ramp exercise test to exhaustion. Cerebral and locomotor muscle oxygenation were measured by means of near infrared spectroscopy. The responses of cerebral and muscle tissue oxygenation index (cTOI, mTOI), oxygenated (O_2_Hb), and deoxygenated hemoglobin (HHb) as a function of work rate were compared. Correlations between residual continuous wave Doppler gradients at rest, arm-leg blood pressure difference and local oxygenation responses were evaluated. Age, length, and weight was similar in both groups. Patients with aortic coarctation had lower peak power output (Ppeak) (72.3 ± 20.2% vs. 106 ± 18.7%, *P* < 0.001), VO_2_peak/kg (37.3 ± 9.1 vs. 44.2 ± 7.6 ml/kg, *P* = 0.019) and %VO_2_peak/kg (85.7 ± 21.9% vs. 112.1 ± 15.5%, *P* < 0.001). Cerebral O_2_Hb and HHb had a lower increase in patients vs. controls during exercise, with significant differences from 60 to 90% Ppeak (O_2_Hb) and 70% to 100% Ppeak (HHb). Muscle TOI was significantly lower in patients from 10 to 70% Ppeak and muscle HHb was significantly higher in patients vs. controls from 20 to 80% Ppeak. Muscle O_2_Hb was not different between both groups. There was a significant correlation between residual resting blood pressure gradient and Δmuscle HHb/ΔP at 10–20W and 20–30W (*r* = 0.40, *P* = 0.039 and *r* = 0.43, *P* = 0.034). Children after coarctation repair have different oxygenation responses at muscular and cerebral level. This reflects a different balance between O_2_ supply to O_2_ demand which might contribute to the reduced exercise tolerance in this patient population.

## Introduction

Repair of coarctation of the aorta (CoA) has been performed for more than 50 years (Cohen et al., 1989; Corno et al., 2001). Although this surgical intervention aims to restore the functional capacity of these patients, exercise performance in this patient group remains impaired in adults (Trojnarska et al., 2007) as well as in children (Rhodes et al., 2010). However, pulmonary gas exchange responses, which should be considered as “whole-body” measurements, obtained during cardiopulmonary exercise tests cannot provide a sufficient insight into the origins of the lower exercise performance. In this context, residual coarctation, left ventricular dysfunction, and hypertension have all been proposed as possible underlying causes. In 1981, Eriksson et al. (Eriksson and Hanson, 1981) observed a disturbed blood flow regulation and impaired blood flow to the working leg muscles during exercise in adults after CoA repair. Johnson et al. (1995) studied blood flow measurements by duplex ultrasound and found an impaired lower limb blood flow in response to strenuous dynamic exercise, even without significant residual stenosis at rest. These studies indicate that also the relationship between O_2_ delivery and O_2_ utilization might be altered in CoA.

Near-infrared spectroscopy is a technique that measures the (changes in) concentration of oxygenated (O_2_Hb) and deoxygenated (HHb) hemoglobin (i.e., tissue oxygenation) during exercise in a non-invasive way. Together with the derived parameter TOI (i.e., tissue oxygenation index: O_2_Hb/(O_2_Hb + HHb) these parameters quantify the overall oxygenation at the level of the tissues. It has been suggested that HHb is a reflection of arterio-venous O_2_ difference (DeLorey et al., 2003; Grassi et al., 2003). According to the principle of Fick, in this context HHb can provide information on the dynamic balance between O_2_ delivery (QO_2_) and O_2_ utilization (VO_2_) at the level of the tissues. In non-steady state conditions there are substantial changes in the ratios between QO_2_ and VO_2_ in different body regions (active/non-active muscles, brain, heart, and other organs, etc.). Therefore, studying the oxygenation patterns at different sites of the body during periods of changing metabolic demand is highly relevant to understanding key aspects of metabolic and vascular control.

At the level of the locomotor muscles HHb increases following a sigmoid pattern as exercise intensity increases during incremental ramp exercise (Boone et al., 2016; Vandekerckhove et al., 2016). The initial slow increase in HHb at the onset of the incremental exercise indicates faster QO_2_ kinetics vs. VO_2_ kinetics at the level of the muscle. In a second phase HHb increases more rapidly revealing an increased fractional O_2_ extraction due to a relative slowing of QO_2_ vs. VO_2_. Finally, a leveling-off in HHb occurs, at an intensity closely corresponding to the respiratory compensation point. Although it has been suggested that fractional O_2_ extraction reaches its limits at this intensity, more recent studies (Inglis et al., 2017; Iannetta et al., 2018) suggest that a local redistribution of blood causes a matching between QO_2_ and VO_2_ with a leveling off in HHb as a consequence.

Also the oxygenation responses at the level of the brain might provide insights into the factors limiting exercise tolerance. It has been shown during incremental ramp exercise that cerebral O_2_Hb increases up to a point at high intensity exercise where a breakpoint occurs and a decline in O_2_Hb is initiated (Bhambhani et al., 2007; Rooks et al., 2010). Cerebral HHb remains stable during submaximal exercise but then shows a rapid increase from hard (>60% $\dot{\text{V}}\,{\text{O}}_{2}$_max_) to maximal intensity (Rooks et al., 2010). These typical O_2_Hb and HHb response patterns indicate that cerebral blood flow increases in accordance with the increase in work rate during incremental exercise (Panerai et al., 1999; Jorgensen et al., 2000; González-Alonso et al., 2004). In a recent study in Fontan patients it was shown that cerebral O_2_Hb did not increase during incremental exercise, which resulted in a progressive decrease in cerebral saturation (i.e., cerebral TOI), as HHb increased during the exercise (Vandekerckhove et al., 2019). These results confirm the potential role of brain oxygenation as a limiting factor to exercise tolerance, especially in patient populations (Brassard and Gustafsson, 2016).

Given the unknown etiology of the lower exercise tolerance in CoA patients after repair, assessing local oxygenation responses (O_2_Hb, HHb, and TOI) during incremental exercise could provide important insights into the underpinning mechanisms. In the past (Eriksson and Hanson, 1981; Johnson et al., 1995) it has been shown that tissue blood flow was affected in individuals following repair of aortic coarctation. Therefore, it can be expected that local oxygenation responses (brain, muscle) during incremental exercise differ from those of healthy controls. Thus, the purpose of the present study was to investigate oxygenation responses at the level of the brain and locomotor muscles during incremental exercise in children after CoA repair. We hypothesize first, that these children will have a lower exercise tolerance compared to healthy children. Second, we hypothesize altered oxygenation responses at the level of the brain and muscle that will be related to residual lesions in the patient group.

## Materials and Methods

### Ethics Statement

This study was approved by the local ethical committee (Ghent University Hospital, Ghent, Belgium) and followed the ethical recommendations for the study of humans as suggested by the Declaration of Helsinki. All participants gave written informed consent prior to the start of the study.

### Participants

Sixteen children post aortic coarctation repair (13 boys, 3 girls) (CoA patients) and twenty healthy children (9 boys, 11 girls) volunteered to take part. The age and anthropometric characteristics of the two groups are presented in Table 1. The groups did not differ significantly for these characteristics. All patients were operated under the age of 4 years, with the majority operated under 1 year (12/16, median 6 weeks, 1 day – 4 years). Most patients (15/16) underwent resection of the coarctation site with end-to-end anastomosis, 1 patient underwent extended arch repair. CoA patients were in stable follow-up. They had normal blood pressures at rest, good left ventricular function (fractional shortening >28% in all patients), and no significant LV hypertrophy on echocardiography. All patients and controls were attending normal school and sports activities.

**TABLE 1:** Age and anthropometric characteristics for coarctation aortae patients and healthy controls.

	**Coarctation**	**Controls**	***P*-value**
Age (years)	13.0 ± 2.2	12.0 ± 1.8	*P* = 0.137
Body weight (kg)	47.5 ± 17.2	41.1 ± 11.0	*P* = 0.104
Body height (m)	1.57 ± 0.13	1.52 ± 0.11	*P* = 0.256
Type of surgery	End to end 15/16		
	Extended end to end 1/16		
Age at surgery (median, min-max)	6 weeks (1 day – 4 years)		
Residual gradient (mm Hg)	26.6 ± 7.3		
LV function (fractional shortening, %)	36.8 ± 5		
Septal thickness (diast)	8.25 ± 1.29		
(*Z*-value)	0.67 ± 0.70		
Posterior wall thickness	7.31 ± 1.49		
(diast) (*Z*-value)	0.55 ± 0.94		

*Values are mean ± *SD*. LV, left ventricle; diast, diastole.*

### Experimental Procedure

An incremental exercise test was performed on an electromagnetically braked cycle ergometer (Ergoline Ergoselect 100K, Bitz, Germany). Following a 3 min warm-up at unloaded cycling, the work rate increased in a linear and continuous way (i.e., ramp exercise). The ramp slope (i.e., the increase in work rate per minute) was individualized and determined by dividing the individual body weight by 4 and rounding off to the closest natural number [0 Watt + (body weight/4) Watt.min^–1^]. This internally validated protocol leads to an optimal exercise duration of 8 – 12 min in healthy children, with reference values equal to the values of Wasserman et al. (Wasserman, 2012). Participants were asked to maintain a pedal rate of 60 revolutions per minute (rpm) and the test was terminated when they reached their self-determined point of full exhaustion or were unable to maintain the required pedal rate despite strong verbal encouragement. Echocardiographic measurements were reviewed and the residual doppler gradient (continuous wave) over the aortic coarctation zone at rest (mmHg) was defined.

### Experimental Measures

During the exercise tests, pulmonary gas exchange (VO_2_, oxygen uptake; VCO_2_, carbon dioxide production; VE, ventilation) was measured continuously on a breath-by-breath basis by means of a computerized O_2_-CO_2_ analyzer-flowmeter combination (Jaeger Oxycon Pro, Germany). Respiratory exchange ratio (RER) was calculated by expressing VCO_2_ relative to VO_2_ (VCO_2_/VO_2_).

Blood pressure in the arm was measured every 3 min during the exercise phase and every 2 minduring the recovery phase with an integrated blood pressure monitor (SunTech Tango) that uses 3D K-Sound Analysis. At the start and at maximal exercise, blood pressure was measured at the leg using the same technique. The difference between systolic pressure arm compared to leg was analyzed at rest and at maximal exercise. This is proven to be an important parameter for the degree of residual obstruction at the aortic arch (Dijkema et al., 2017).

Muscle and cerebral oxygenation (O_2_Hb and HHb) were measured by means of near infrared spectroscopy technology (NIRO-200NX, Hamamatsu Photonics K.K., Hamamatsu, Japan). This system consists of an emission probe emitting near-infrared light at three wavelengths (735, 810 and 850 nm) and a photon detector which measures the intensity of incident and transmitted light at a frequency of 2 Hz. For measurements of oxygenation, the probe was positioned longitudinally over the distal section of the left M. Vastus Lateralis and adhered to the skin. For measurements of cerebral oxygenation, the probe was placed over the left pre-frontal lobe, approximately 3 cm from the midline and just above the supra-orbital ridge (Kleinschmidt et al., 1996; Bhambhani et al., 2007). This device measures TOI as a reflection of mixed arterio-venous O_2_ saturation (in%) at the location of the probe. Additionally, relative changes to baseline values in the concentration of O_2_Hb and HHb (in μmol) are recorded. Baseline cycling at 0 Watt was used as baseline values for O_2_Hb and HHb and were set to 0 μmol.

### Data Analysis

#### Cardiopulmonary Exercise Test

The breath-by-breath data from the gas exchange responses were filtered upon exportation based on the following criteria: tidal volume < 0.2 and >10 l⋅min^–1^; fraction of expired CO_2_ <1 and >10% (Fontana et al., 2015). The VO_2_peak was calculated as the highest 30s average (i.e., moving average) VO_2_ throughout the test. Since a leveling-off in VO_2_ is often not reached in children (Armstrong and Welsman, 1994), the term VO_2_peak will be used throughout to avoid erroneous conclusions on maximal effort. The peak power output (Ppeak) was determined as the work rate attained at the termination of the exercise phase. The VO_2_peak and Ppeak were expressed relative to the norm values (predVO_2_peak, predPpeak), based on age and anthropometrics (Wasserman, 2012).

The gas exchange threshold (GET) was determined using the criteria of a disproportionate increase in carbon dioxide production (VCO_2_) to VO_2_ (Beaver et al., 1986), a first departure from the linear increase in minute ventilation (VE) and an increase in VE/VO_2_ with no increase in VE/VCO_2_. The disproportionate increase in VCO_2_ is related to an increase in the buffering of H^+^ due to an increased production of pyruvate from glycolytic processes in the cytosol of muscle fibers. The peak Respiratory Exchange Ratio (RERpeak) was determined as the highest 30s RER throughout the test, the peak heart rate (HRpeak) as the highest value obtained throughout the test.

#### Cerebral and Muscle Oxygenation

The changes in TOI and in the concentration of cerebral and muscle O_2_Hb and HHb from baseline values (i.e., baseline cycling at 0 Watt) of each individual were expressed as a function of Ppeak by calculating the mean TOI, O_2_Hb and HHb response at 10%, 20%, 30%, …, 100% Ppeak. The values at these% Ppeak were calculated as the average of the O_2_Hb and HHb values 10s prior and 10s following the relative intensity.

Additionally, to quantify the relationship between muscle O_2_ supply and O_2_ demand, the change in muscle HHb (Δmuscle HHb) as a function of the change in work rate (ΔP) (Δmuscle HHb/ΔP) for each 10% Ppeak interval (i.e., from 0 to 10%, 10 to 20%, *etc*) was calculated.

To quantify the sudden changes in the pattern of the NIRS responses, breakpoints (BP) were determined. Therefore, the studies of Miura et al. (1998) and Spencer et al. (2012) were used as examples of typical responses in muscle O_2_Hb (BP at moderate and high intensity demarcating the point of an accelerated and attenuated decrease, respectively) and HHb (BP at high intensity at which muscle HHb levels off), respectively. The studies of Rooks (Rooks et al., 2010) and Bhambani (Bhambhani et al., 2007) served as examples of the typical responses in cerebral O_2_Hb (BP at high intensity at which O_2_Hb starts to decrease) and HHb (BP demarcating the point of an accelerated increase). In case the determination of the breakpoints was not possible with the two-segment linear piecewise model of curve fitting in Sigmaplot (Systat Software Inc., San José, CA, United States), two experienced researchers analyzed the oxygenation responses visually to detect the BPs. When the analysis did not correspond between the two researchers, the data were re-evaluated together with a third researcher until a consensus was reached.

Finally, the amplitude of the NIRS responses was calculated as the difference between the NIRS value at baseline cycling and the highest (or lowest) obtained value throughout the test (i.e., either at the BP or Ppeak).

### Statistical Analysis

The statistical analysis was performed in SPSS 21.0 (IBM Corp., Armonk, NY, United States). The pulmonary gas exchange (VO_2_, VCO_2_) and NIRS (TOI, O_2_Hb, HHb) data were normally distributed, and therefore the data are presented as mean values ± SD and parametric statistical analyses were performed. The parameters quantifying exercise tolerance (Ppeak, VO_2_peak, and GET) were compared between the CoA patients and healthy controls by means of Independent Samples *T*-tests. The predPpeak and predVO_2_peak in both patients and controls were compared to a reference value of 100% (Wasserman, 2012) by means of One Sample *T*-tests. The cerebral and muscle TOI, O_2_Hb, and HHb responses at the 10% Ppeak intervals (between 0% and 100% Ppeak) were compared between the CoA patients and healthy controls and between the intensities by means of Two-way Anova (Group x Intensity). Additionally, the Δmuscle HHb/ΔP values were compared at each relative intensity (% Ppeak) between patients and controls by means of Two-way Anova. In case of significant interaction or main effects *post hoc* Tukey tests were performed. Statistical significance was set at *P* < 0.05.

## Results

### Exercise Tolerance

In Table 2 the parameters quantifying exercise tolerance, obtained from the incremental ramp exercise, are presented. When expressed relative to body weight, the CoA subjects had significantly lower Ppeak (Watt.kg^–1^) (*P* = 0.010), VO_2_peak (ml.min^–1^.kg^–1^) (*P* = 0.019), and GET (ml.min^–1^.kg^–1^) (*P* = 0.002). Also, the CoA patients had significantly lower Ppeak and VO_2_peak values compared the expected values for age, gender, stature and weight (*P* < 0.001).

**TABLE 2:** Exercise tolerance (Ppeak, VO_2_peak, RERpeak, HRpeak, GET, and blood pressure) in coarctation aortae patients and healthy controls,

	**Coarctation**	**Controls**	***P*-value**
Ppeak (Watt)	119 ± 49	125 ± 41	*P* = 0.723
Ppeak/kg (Watt.kg^–1^)	2.42 ± 0.65	3.04 ± 0.59	*P* = 0.010^∗^
% Predicted Ppeak (%)	72.3 ± 20.2	106 ± 18.7	*P* < 0.001^∗^
VO_2_peak (ml.min^–1^)	1792 ± 581	1790 ± 459	*P* = 0.991
VO_2_peak/kg (ml.min^–1^.kg^–1^)	37.3 ± 9.1	44.2 ± 7.6	*P* = 0.019^∗^
% Predicted VO_2_peak (%)	85.7 ± 21.9	112.1 ± 15.5	*P* < 0.001^∗^
RERpeak	1.14 ± 0.10	1.09 ± 0.06	*P* = 0.382
HRpeak (bts.min^–1^)	179 ± 19	193 ± 9	*P* = 0.188
GET (ml.min^–1^)	933 ± 371	964 ± 289	*P* = 0.657
GET/kg (ml.min^–1^.kg^–1^)	19.0 ± 4.7	23.6 ± 3.9	*P* < 0.002^∗^
Blood pressure rest (sys/dias) (mm hg)	121 ± 19/68 ± 12	110 ± 16/63 ± 16	*P* = 0.561/*P* = 0.624
Blood pressure max (sys/dias) (mm hg)	175 ± 22/69 ± 10		

*Values are mean ± *SD*. Ppeak, peak power output; $\dot{V}$O_2_peak, peak oxygen uptake; RERpeak, peak respiratory exchange ratio; HRpeak, peak heart rate; GET, gas exchange threshold; BP, blood pressure. ^∗^Indicates a significant (*P* < 0.05) difference between coarctation and controls.*

### Cerebral and Muscular Oxygenation

**FIGURE 1:**
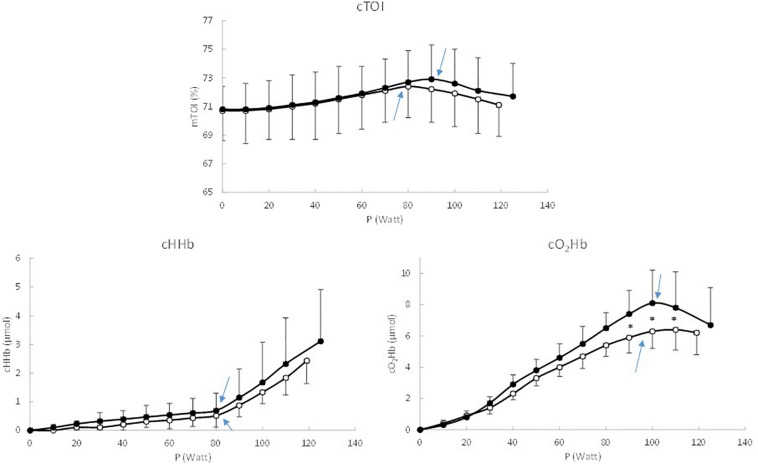
Response pattern of cerebral TOI, HHb, and O_2_Hb as a function of work rate, expressed in 10% Ppeak intervals. Black circles represent the healthy controls, white circles represent the coarctation aortae patients, and ^∗^ indicate significant differences between patients and controls.

**FIGURE 2:**
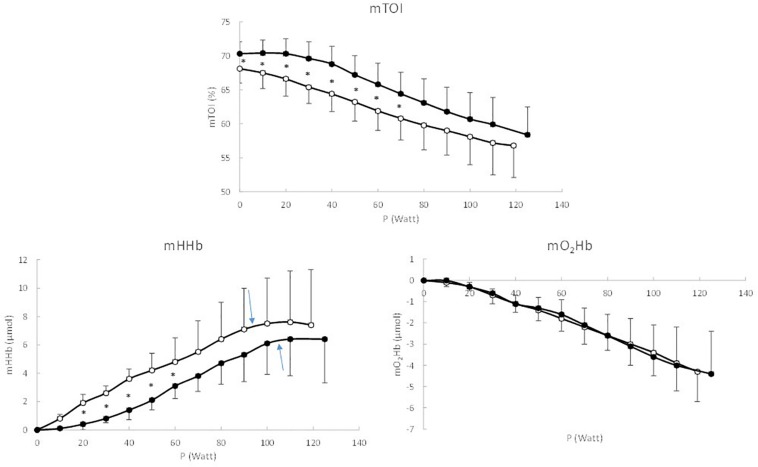
Response pattern of muscle TOI, HHb, and O_2_Hb as a function of work rate, expressed in 10% Ppeak intervals. Black circles represent the healthy controls, white circles represent the coarctation aortae patients, and ^∗^ indicate significant differences between patients and controls.

In Figures 1, 2, the cerebral and the muscular TOI, HHb and O_2_Hb patterns are presented as a function of intensity in 10% Ppeak intervals. For cerebral TOI there were no differences between patients and controls (*P* > 0.05) at any intensity. However, the BP in cerebral TOI (at which TOI starts to decrease) occurred at a significantly lower absolute (78 ± 33 Watt vs. 93 ± 37 Watt, respectively, *P* = 0.027) and relative intensity (65.5 ± 9.7% Ppeak vs. 74.4 ± 11.2% Ppeak, respectively, *P* = 0.014) in patients compared to controls.

For the cerebral O_2_Hb (*P* = 0.038) and HHb (*P* = 0.045) a significant interaction effect (Intensity x Group) was demonstrated, indicating that the response pattern differed between CoA patients and healthy controls. For cerebral O_2_Hb, *post hoc* tests revealed that cerebral O_2_Hb was significantly lower (*P* < 0.05) from 60 to 90% Ppeak in patients vs. controls. Also the BP occurred at a significantly lower absolute (89 ± 36 Watt vs. 101 ± 35 Watt, respectively, *P* = 0.027) and but not relative intensity (74.8 ± 12.1% Ppeak vs. 80.8 ± 9.4% Ppeak, respectively, *P* = 0.102) in patients compared to controls. For cerebral HHb, *post hoc* tests showed that the increase in cerebral HHb was more pronounced in healthy controls from 70 to 100% Ppeak. The BP did not occur at a different absolute (78 ± 36 Watt vs. 78 ± 32 Watt, respectively, *P* = 0.027) and relative intensity (66.0 ± 11.8% Ppeak vs. 66.8 ± 10.4% Ppeak, respectively, *P* = 0.862) in patients and controls.

For muscle TOI a significant main effect (*P* = 0.021) of Group was found. *Post hoc* analysis showed that muscle TOI was significantly (*P* < 0.05) lower in patients compared to controls from 10 to 70% Ppeak. The total amplitude of the decrease in muscle TOI did not differ significantly (*P* = 0.739) between both groups (−11.7 ± 4.6% vs. −11.6 ± 5.5% in patients and controls, respectively) and also the muscle TOI at Ppeak (56.8 ± 4.0% vs. 58.4 ± 5.1% in patients and controls, respectively did not differ significantly (*P* = 0.372). Also for muscle HHb a significant (*P* = 0.010) main effect of Group was found. Muscle HHb was significantly (*P* < 0.05) higher in patients compared to controls from 20 to 70% Ppeak. The BP in muscle HHb occurred at a significantly lower absolute (92 ± 33 Watt vs. 105 ± 36 Watt, respectively, *P* = 0.031) and relative intensity (77.1 ± 9.1% Ppeak vs. 84.4 ± 9.7% Ppeak, respectively, *P* = 0.039) in patients compared to controls, whereas the maximal amplitude of the muscle HHb response (7.6 ± 3.6 μmol vs. 6.8 ± 3.6 μmol, respectively, *P* = 0.466) did not differ significantly between patients and controls.

**FIGURE 3:**
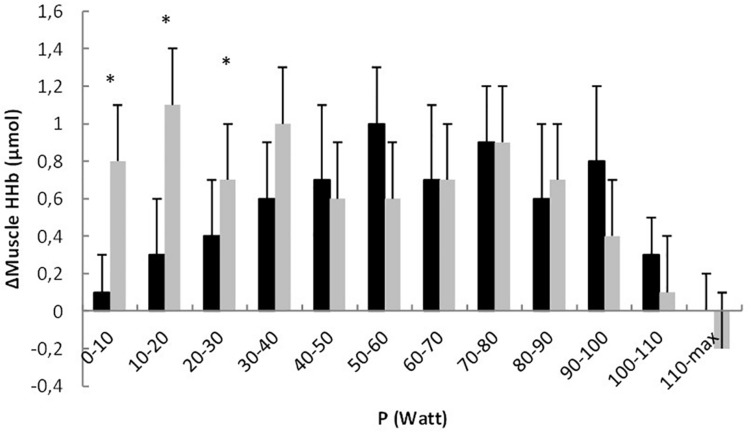
Change in muscle HHb (Δmuscle HHb) relative to the change in work rate (ΔP) for each 10 Watt interval in healthy controls (black bars) and coarctation aortae patients (gray bars). ^∗^ indicate significant differences between the groups.

In Figure 3 Δmuscle HHb is presented for each 10 Watt increase. It was observed that Δmuscle HHb was significantly higher in CoA patients compared to healthy controls for the 0–10, 10–20, and 20–30, 30–40 Watt intervals (*P* < 0.05). Muscle O_2_Hb did not differ significantly (*P* > 0.05) between CoA patients and controls over the entire intensity range and at Ppeak (−4.3 ± 1.4 μmol vs. −4.4 ± 2.0 μmol, respectively, *P* = 0.792). A clear BP could only be found in 4 of 16 CoA patients and 7 of 20 controls, therefore the BP in muscle O_2_Hb was not considered.

**FIGURE 4:**
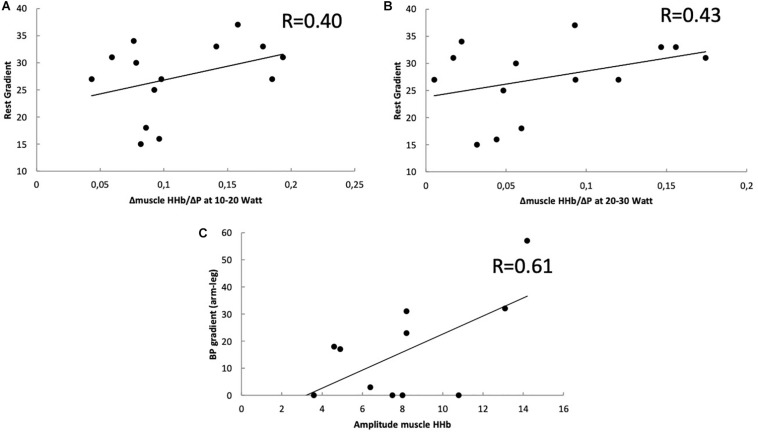
Correlation between the change in muscle HHb (Δmuscle HHb) and residual echocardiographic **(A,B)** or blood pressure gradient **(C)**. **(A)** Relative to the change in work rate (ΔP) for 10–20 Watt. **(B)** Relative to the change in work rate (ΔP) for 20–30 Watt **(B)** Ppeak and the residual gradient using echocardiography in coarctation patients. **(C)** The correlation between the total amplitude of muscle HHb (difference between 0% Ppeak and 100% Ppeak) and blood pressure difference between arm and leg at maximal exercise.

Finally, it was also found that some NIRS variables were correlated to clinical indices. ΔMuscle HHb for the 10–20 and 20–30 Watt intervals (10–20 Watt: *r* = 0.40; *P* = 0.039 and 20–30 Watt: *r* = 0.43; *P* = 0.034) showed a weak but significant correlation with the residual gradient over the coarctation zone (Figure 4). The total amplitude of muscle HHb (i.e., the change between 0% Ppeak and 100% Ppeak) was significantly correlated (*r* = 0.61, *P* = 0.017) to the blood pressure difference between the arm and leg at maximal exercise.

## Discussion

To the best of our knowledge, this is the first study to report the patterns of cerebral and muscular tissue oxygenation during incremental exercise in children with aortic coarctation. It was found that children with CoA had a lower exercise tolerance, as can be deducted from a lower VO_2_peak, Ppeak and GET, expressed relative to body weight. Additionally, there are different oxygenation patterns at the cerebral and muscle level compared to healthy children. More specifically, children with CoA had a less pronounced increase in cerebral O_2_Hb at high intensities, whereas muscle TOI and HHb showed a more rapid decrease and increase, respectively, especially at low to moderate exercise intensities. Also the breakpoints in cerebral TOI and muscle HHb occurred at a lower absolute and relative intensity, indicating that peripheral oxygenation might contribute to the lower physical fitness levels observed in CoA patients compared to healthy controls.

### Exercise Performance

In accordance with previous studies (Trojnarska et al., 2007; Hager et al., 2008), we found a lower exercise performance in patients after CoA repair. The CoA patients reached only 72.3 ± 20.2% of the predicted Ppeak and 85.7 ± 21.9% of the predicted VO_2_peak, which was significantly lower compared to the healthy subjects. The GET, quantifying aerobic exercise tolerance, was also lower in CoA patients.

With regards to the potential underpinning mechanisms of lower exercise performance in CoA patients is has been suggested that there might be a reduced aortic compliance after CoA repair (Hager et al., 2008). Additionally, also a reduced cardiac output could potentially contribute to the reduced exercise performance. Left ventricular hypertrophy in patients with residual CoA can disturb diastolic function with a decreased cardiac output at high intensities. However, at the moment there is no scientific evidence of an affected cardiac output in CoA patients. The present study indicates that different flow distribution patterns and local changes in oxygenation could also contribute to diminished exercise performance (see below). The observation that children after CoA repair appear to have an earlier reliance on the anaerobic metabolism (Wong et al., 2017) supports the suggestion of a reduced functional capacity of the aerobic metabolism. However, at the moment the main origin of the limitation (i.e., convective O2 supply, O_2_ diffusive capacity) is unknown.

Additionally, it should be noted that next to underlying pathophysiological factors also deconditioning in relation to lower physical activity (due to an overprotective environment) might be a possible contributing factor to the lower exercise performance. However, a recent study of Stone et al. (2015) showed that the physical activity levels of CoA children after repair were similar to those of healthy children.

### Cerebral Oxygenation

The lower exercise tolerance in CoA patients could at least in part be explained by a different oxygenation pattern at the peripheral level. Similar to healthy subjects (Rooks et al., 2010; Vandekerckhove et al., 2016) cerebral O_2_Hb increased from low to high intensities, where a breakpoint occurred at which cerebral O_2_Hb levels off or even decreased. Cerebral HHb showed a slow initial increase with a progressive speeding as work rate increased (>60% Ppeak). In comparison to the healthy controls the amplitude of the responses was less pronounced for cerebral O_2_Hb. The combination of both a lower cerebral O_2_Hb and a similar HHb in CoA patients vs. controls at high intensity explains the lower cerebral TOI (i.e., mixed arterio-venous saturation) in CoA patients at high intensities. It is unclear what might be at the origin of this different oxygenation pattern at cerebral level. One study, although not in coarctation patients, reported that cerebral hemodynamics adapt very rapidly to changes in tension when hypertensive adult patients received antihypertensive medication (Zhang et al., 2007). A recent study described an increased intracranial arterial stiffness and decreased responsiveness to hypercapnic stimuli in adult CoA patients (Wong et al., 2017).

Patients after coarctation repair might suffer from residual narrowing and/or arterial stiffnes distal from the origin of the arteries supplying blood flow to the brains. This leads to a higher pressure and hypertension at the level of the brain. How the brain copes with higher pressures during exercise, and if an (over)protective mechanism is more active in children with CoA, is not known.

The results of the present study show a less pronounced increase in O_2_ supply (as reflected in the O_2_Hb response), in combination with an earlier onset of the decrease (i.e., the breakpoint) in cerebral O_2_Hb which resulted in an earlier decrease in cerebral TOI in CoA patients compared to controls. It can be speculated that this affected the “activity” of the motor cortex as such that the firing rate to the locomotor muscles was reduced which might have resulted in an earlier termination of the exercise test. In a recent study in our laboratory, it was observed that Fontan children had a fast decrease in cerebral TOI from the onset of the incremental ramp exercise (Vandekerckhove et al., 2019) and terminated the test with a reduced cerebral TOI compared to healthy controls. In this population it was speculated that the cerebral oxygenation might have been the main contributing factor to exercise termination. Whether this is also the case in the CoA children is questionable since cerebral TOI at Ppeak was similar to the rest values and did also not differ with the controls.

#### Muscle Oxygenation

Also, the oxygenation at the locomotor muscles showed a different pattern in the two study groups. In the CoA patients, muscle HHb showed a more pronounced increase in the low to moderate intensity domain in combination with a lower TOI compared to healthy controls. As HHb is often considered as the most valuable NIRS parameter since it is a reflection of fractional O_2_ extraction (McNarry et al., 2015), this different pattern of HHb might reflect a disturbed relationship between O_2_ supply and O_2_ demand. In healthy subjects the HHb response to incremental ramp exercise shows a sigmoidal pattern (Carano et al., 1999; Boone et al., 2009; Vandekerckhove et al., 2016) with a rather slow increase in HHb at the onset of the incremental ramp exercise. This typical pattern in the HHb response at low to moderate intensities indicates that the blood flow to the locomotor muscles has increased to such an extent that the fractional O_2_ extraction does not need to increase (Ferreira et al., 2007; Boone et al., 2009). In the CoA patients however, this “sigmoidal” pattern is not present and the HHb response shows an immediate increase at the onset of exercise (Figure 2). This more pronounced increase in HHb in CoA patients is also expressed in Figure 3. This indicates that the balance between O_2_ supply and O_2_ demand might be altered at low to moderate intensities, highly likely related to a disturbed convective O_2_ delivery. This disturbed balance at low intensities is also reflected in the lower mTOI values during unloaded cycling.

Interestingly, we found a correlation between residual arch gradient and ΔmuscleHHb/ΔP and between the total muscle HHb amplitude and blood pressure difference arm-leg at maximal exercise. The CoA children with higher residual obstruction at the descending aorta, have more pronounced increase in muscle HHb per increase in work rate and thus a more disturbed balance between O_2_ supply and O_2_ demand. Exercise testing has been shown to be a useful tool for evaluation of residual coarctation after surgery (Carano et al., 1999; Das et al., 2009). Correia et al. (2013) showed that patients with higher residual gradient can develop hypertension during exercise, despite normal tension control at rest. A difference in exercise capacity between adults with higher residual gradient or normal gradient could not be shown (Trojnarska et al., 2007), although exercise capacity is generally decreased in patients after CoA repair. The different mechanisms at the level of the muscles are unknown. Our findings demonstrate that there might also be metabolic differences at the muscular level in children after CoA repair, even more pronounced in children with higher residual stenosis. Surprisingly, this study also showed that CoA patients have a higher total amplitude of the muscle HHb response compared to healthy subjects, indicating a greater O_2_ extraction capacity in this patient population. It is highly likely that the greater reliance on O_2_ extraction, due to the disturbed balance between O_2_ supply and O_2_ demand even at low to moderate intensities, results in the greater capacity of the locomotor muscles to extract O_2_.

Although the limited number of patients should be considered a limitation of the present study, the results shed a new light on the exercise tolerance of CoA patients. The evaluation of cerebral and peripheral oxygenation in this patient population provides useful information on the physical condition of the subjects and the efficacy of treatments and rehabilitation programs. Larger patient trials are needed to explore the influencing factors and causes which can possibly explain the different patterns in CoA patients. In this context, it would be useful to integrate NIRS measurements to assess whether the oxygenation patterns could provide a more comprehensive insight into the exercise performance of CoA patients.

### Conclusion

Children after coarctation repair have diminished exercise capacity in combination with different patterns of oxygenated and deoxygenated hemoglobin at the level of the brains and at the muscular level. This points toward diminished blood flow and oxygen transport at the level of the brains and increased oxygen extraction at the level of the muscles during exercise. The increased muscular deoxygenation is more pronounced in children with higher residual coarctation gradient and blood pressure gradient. The measurement of peripheral oxygenation during exercise might provide useful information with regards to the disease state of the individual patient.

## Data Availability Statement

The datasets generated for this study are available on request to the corresponding author.

## Ethics Statement

The studies involving human participants were reviewed and approved by the Ghent University Hospital. Written informed consent to participate in this study was provided by the participants’ legal guardian/next of kin.

## Author Contributions

KV, IC, DD, and JB contributed to the study design. KV, IC, JP, AM, KD, and TB contributed to the data collection. KV, IC, AM, TB, HD, KF, and JB contributed to the data analysis. KV, IC, TB, DD, AM, and JB contributed to the data interpretation. KV, IC, JP, KD, and JB contributed to the writing of the manuscript. AM, KD, TB, JP, HD, and KF contributed to the revision of the manuscript.

## Conflict of Interest

The authors declare that the research was conducted in the absence of any commercial or financial relationships that could be construed as a potential conflict of interest.
